# Spatial Structure of Lectin from the Mussel *Mytilus trossulus*: In-Sights from Molecular Modelling and Practical Proof

**DOI:** 10.3390/md21010010

**Published:** 2022-12-23

**Authors:** Alina P. Filshtein, Irina V. Chikalovets, Tatyana O. Mizgina, Pavel A. Lukyanov, Kuo-Feng Hua, Oleg V. Chernikov

**Affiliations:** 1 G.B. Elyakov Pacific Institute of Bioorganic Chemistry FEB RAS, Vladivostok 690022, Russia; 2 Department of Biotechnology and Animal Science, National Ilan University, Ilan 260007, Taiwan; 3 Department of Medical Research, China Medical University Hospital, China Medical University, Taichung 404327, Taiwan

**Keywords:** lectin, Mytilectin, dimerization, β-trefoil fold, molecular modeling

## Abstract

Most proteins have the ability to self-associate into homooligomeric protein complexes, which consist of two or more identical subunits. Today, modern methods of molecular modeling are an integral part of the study of many biologically active molecules. In silico methods are widely used in structure establishing and function and activity prediction of lectins – carbohydrate-binding proteins. Here, we described by computer simulation the spatial organization of lectin isolated from the mantle of the mussel *Mytilus trossulus* (MTL). It was shown that the dimerization of MTL gives a total of six ligand binding sites that may be important for the manifestation its biological properties. The ability of MTL to form a dimeric and oligomeric structure was confirmed by dynamic light scattering and SDS-PAGE methods.

## 1. Introduction

Lectins are carbohydrate-binding proteins that comprise a non-catalytic domain that can bind with specific carbohydrate moieties, i.e. monosaccharides and oligosaccharides [1]. Lectins are crucial in the specificity of molecular recognition in various physiological processes and are already considered a standard for determining protein–carbohydrate interaction [2]. Carbohydrate–protein interactions compose the basis of different mechanisms that are necessary for both extracellular and intracellular physiological processes, such as cell adhesion, cell-to-cell communication, signaling and transport, and glycoprotein folding [3]. Owing to the recognition of endogenous carbohydrate fragments, lectins are involved and associated with embryogenesis, tissue development and regeneration, and the regulation of immune system functions [4,5,6]. Contrariwise, lectins can function as critical factors in the establishment and maintenance of highly specific mutualistic associations in host–microbe complexes, as well as in innate immunity responses to infection by interacting with foreign exogenous glycoconjugates on the surface of pathogenic microorganisms [3,4]. Despite the high degree of specificity of lectins for glycan structures, their binding affinity for a single site is usually low. In biological systems, functional affinity is most commonly achieved by oligovalent presentation of carbohydrate-recognition domains (CRDs) through protein oligomerization [7]. Multivalence is a peculiarity of lectins, which provides an increase in the affinity of lectins when interacting with ligands [8]. An increase in affinity for a ligand can be achieved by clustering several similar binding domains, followed by oligomerization of polypeptides, for example, C-type lectins [9]. Some lectins, such as CGL, form oligomers and achieve high affinity for carbohydrates due to internal duplications of several independent binding sites in a single polypeptide chain [8]. CGL, a lectin isolated from the mussel *Crenomytilus grayanus*, belongs to a recently formed Mytilectin family, which, until recently, included lectin from the mussel *Mytilus galloprovincialis* (MytiLec) [10] and lectin from the mussel *M. californianus* (MCL) [11]. 

Interest in this family is due to the fact that despite the high identity of proteins in structure (identity reaches 85%) and physicochemical characteristics, they are able to have some effects on different types of tumor cells and pathogenic microorganisms [11,12]. The presence of the dimeric quaternary structure of these lectins and the doubling of the ligand-binding site lead to the formation of a total of six ligand-binding sites within the dimer [13], which provides them with high avidity with respect to large ligands, since the affinity of individual carbohydrate-binding sites of lectins is rather weak [14]. 

MTL, lectin from the mussel *M. trossulus*, is another representative of the Mytilectin family [12,15]. Since there is evidence that dimerization of the molecules is a necessary condition for the manifestation of the biological activity of these lectins [12], in this work, we investigated the ability of MTL to self-associate with the formation of dimers and oligomers.

## 2. Results

### 2.1. Multiple Alignments of Mytilectin Family Lectins

**Figure 1:**
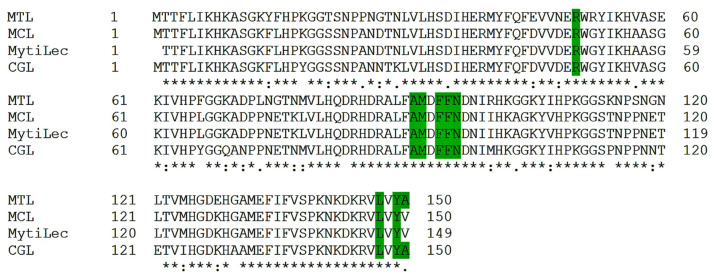
Comparative analysis of amino acid sequences of lectins of the Mytilectin family. The amino acids responsible for the formation of the dimeric structure are highlighted in green.

According to phylogenetic analysis MTL has a close evolutionary relationship with lectins of the Mytilectin family [11]. Alignment of amino acid sequences showed a high percentage of identity; more than 85%. It was also found that MTL contains amino acids that contribute to the formation of a dimeric structure and its stabilization, as in CGL (Ala91, Met92, Phe94, Phe95, Leu147, Tyr149, Ala150) [13], MCL (Arg50, Phe94, Phe95, Asn96) [11] and MytiLec (Phe94, Phe95) [16] (Figure 1).

Thus, it can be assumed that MTL is able to form a stable dimeric structure due to the presence of Arg50, Ala91, Met92, Phe94, Phe95, Asn96, Leu147, Tyr149, Ala150 in its amino acid sequence.

### 2.2. Molecular Modeling of the MTL Structure

The theoretical model of MTL spatial structure was constructed using the SWISS-MODEL server (https://swissmodel.expasy.org/interactive (accessed on 1 March 2022)) containing the template library. As a result, the eight most suitable protein prototypes with an already known crystal structure were found for the target MTL sequence (Table 1). Template search has been performed against the SWISS-MODEL template library (SMTL, last update: 14 September 2022, last included PDB release: 9 September 2022). For each identified template, quality was predicted from the features of the target–template alignment. The templates with the highest quality have been selected then for model building. Models were built based on the target–template alignment using ProMod3 [17]. Coordinates, which are conserved between the target and the template, were copied from the template to the model. Insertions and deletions were remodeled using a fragment library. Side chains were then rebuilt. Finally, the geometry of the resulting model was regularized by force field. The global and per-residue model quality was assessed using the QMEANDisCo scoring function [18].

The QMEANDisCo global score for each prototype can be used to determine the quality of the constructed theoretical protein model both globally (for the entire construct) and locally (for individual residues). In other words, the QMEANDisCo score shows how the constructed model is comparable with the experimental structures of proteins of the same size. A QMEANDisCo score close to 1.0 indicates good agreement between the theoretical model and experimental structures of the same size. Based on the data presented in Table 1, it was found that the most accurate models of the MTL dimer structure are the 3wmu.1.B, 3wmu.1.A, and 5f8s.1.B models.

**Figure 2:**
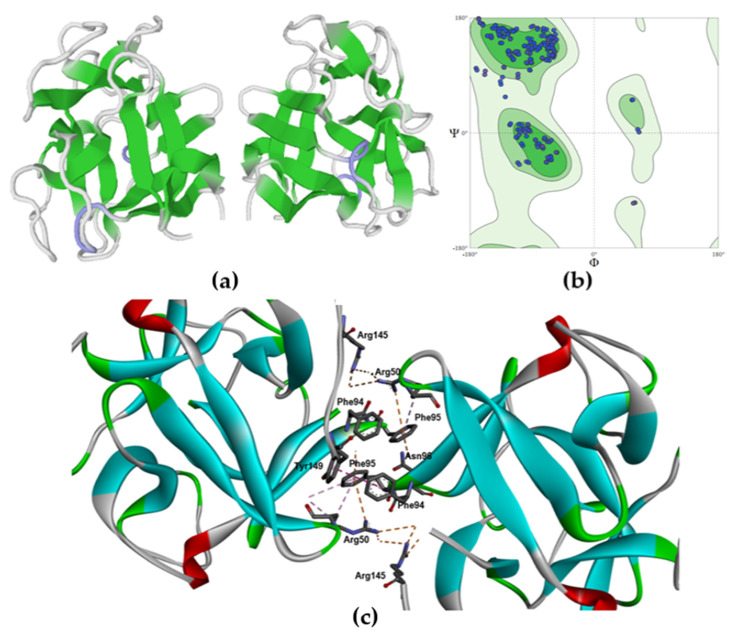
Molecular modeling of the MTL structure: (**a**) homology modelling the dimer of the lectin by SWISS-MODEL (https://www.swissmodel.expasy.org/ (accessed on 1 March 2022)); (**b**) Ramachandran plot for the validation of the lectin model; (**c**) theoretical model of the spatial structure of the MTL dimer, con-structed in the Discovery Studio Visualizer software (the secondary structure of the protein is identified by color: blue—β-sheet, white—coil, green—turn and red—α-helix).

The presented lectins belong to the Mytilectin family and have high amino acid sequence identity with MTL. Therefore, the construction of a model of the MTL dimeric structure was carried out using the CGL homodimer (PDB identifier: 5f8s.1.B) as a prototype. (Figure 2a). The stereochemical quality and reliability of the structure were tested using the Ramachandran plot and Z-score. As shown in Figure 2b, most amino acid residues (96.6%) are in the favorable region on the Ramachandran plot. The Z-score of the protein was calculated to be -5.3, which is in the range of scores commonly found for native proteins of similar size [19].

The resulting lectin model was visualized using BIOVIA Discovery Studio Visualizer (Figure 2c). It has been established that the dimeric structure of MTL is formed as a result of folding and intercalation of the aromatic side chains of Phe94, Phe95, and Tyr149, followed by stabilization of the structure through hydrogen bonds and hydrophobic interactions of the following amino acids: Glu49, Arg50, Asp93, Asn96, and Ala150. The result obtained correlates with the results described for the crystal structures of the CGL [13], MCL [11] and MytiLec [16] homodimers.

### 2.3. Practical Proof of MTL Oligomerization

Dynamic light scattering (DLS), being highly sensitive to large sizes, is the best method for detecting aggregation. Protein samples can display inter-particle and particle–solvent interactions that can cause substantial distortion in the apparent diffusion coefficients determined by DLS, and hence, affect the calculated hydrodynamic size [20]. Correlating the dynamic radius of a protein with its molecular weight seems to be a rather difficult task, since each protein has its own charge and spatial structure. DLS is based on the scattering of light from particles and their inherent Brownian motion.

**Figure 3:**
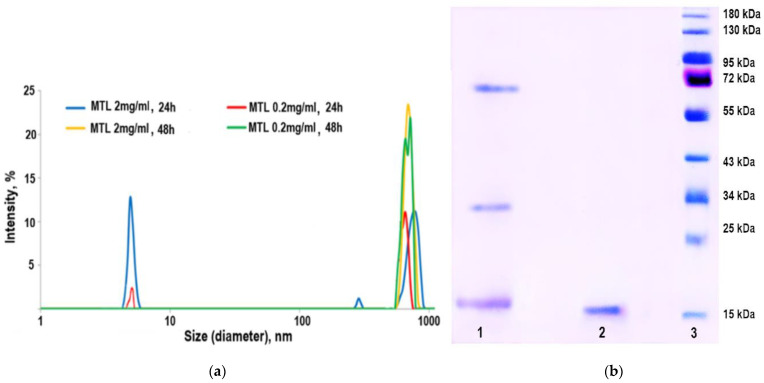
Practical proof of MTL oligomerization: (**a**) size distribution of MTL particles for solutions with different concentrations at different time intervals, obtained by the DLS method; (**b**) electropherogram of MTL samples depending on the exposure time in solution, obtained by SDS-PAGE: 1—MTL after exposure for 48 days, 2—freshly isolated MTL, 3—molecular weight standards.

DLS and SDS-PAGE (Figure 3) were used to study the dynamics of MTL behavior in solutions with different lectin concentrations. 

Figure 3 shows the size distribution of lectin particles in solution depending on the protein concentration at different time intervals. MTL solutions of both concentrations after 24 h were shown to contain monomeric particles with a diameter of about 4.8 nm and large aggregates. After 48 h, we observed only aggregates. The high tendency to oligomerization is due to the presence of a large number of amino acids capable of forming and stabilizing aggregates. The obtained results are consistent with the data of SDS-PAGE. With an increase in the concentration of MTL or the time of its exposure in solution, lectin dimerization occurs, which subsequently leads to its oligomerization (Figure 3).

## 3. Discussion

It is known that in the first publications devoted to the isolation of lectins of the Mytilectin family, they were described as monomers [16,17,18,19,21,22,23]. Further experiments made it possible to state with confidence that dimerization is a necessary condition for the manifestation of their biological activity [16]. Moreover, CGL and MCL formed high molecular oligomers as the concentration increased. Using MALDI-TOF mass spectrometry, the minimum concentration of CGL self-association beginning was determined. A small amount of tetramer appeared already at a concentration of 0.1 mg/mL [23]. MCL could not be concentrated above 2 mg/ml because it tended to be highly oligomerized [11]. It is known that oligomerization contributes to the structural stabilization of lectins and increases their functionality. MTL, like other members of the Mytilectin family, contains three structurally conserved subdomains forming a β-trefoil fold [15]. An important conclusion from our study is that the dimeric spatial structure and doubling of ligand binding sites allows MTL to give a total of six ligand binding sites to the lectin, that may be important for the manifestation its biological properties. As evidence, Terada et al. demonstrated that Mitsuba-1, a mutant protein based on MytiLec, cannot form dimers, which leads to a loss of the ability to agglutinate and cytotoxicity towards tumor cells [14]. 

Mytilectin family lectins are unusual among natural β-trefoil lectins in that each sequence repeat forms a sugar-binding site so that each polypeptide binds three identical ligands, whereas β-trefoil lectins bind only one ligand per protein subunit. Lectins with tandem repeats in their structure, folding into β-trefoils, are good candidates as biomarkers due to their high avidity to glycans located on cell membranes. By resorting to engineering strategies such as point mutation, inclusion of non-canonical amino acids, or directed evolution, it is now possible to design and control the specificity and topology of such lectins. The development of such multivalent receptors using artificially designed tandem repeats is of interest, since this greatly increases the affinity of the receptor for its ligand [24].

## 4. Materials and Methods

### 4.1. MTL Purification

Lectin from the mussel *Mytilus trossulus* was isolated by affinity chromatography on porcine stomach mucin (PSM)-Sepharose 4B (GE Life Sciences, Uppsala, Sweden) and following gel permeation chromatography on Sephacryl S-200 column (GE Life Sciences, Uppsala, Sweden), as described in [15].

### 4.2. Sodium Dodecyl Sulfate-Polyacrylamide Gel Electrophoresis (SDS-PAGE)

SDS-PAGE was carried out by Laemmli [25] using an EPS 301 power supply (Amersham Pharmacia Biotech, Uppsala, Sweden) and separating (12.5%) and concentrating (4%) polyacrylamide gel (PAAG). The molecular weight of lectin was determined using a mixture of ten pre-stained recombinant proteins of known molecular weight (Thermo Fisher Scientific, Vilnius, Lithuania).

### 4.3. Dynamic Light Scattering Method

DLS measurements were performed with a Malvern Zetasizer Nano ZS (Malvern, Herrenberg, Germany) equipped with a 633-nm He-Ne laser and operating at an angle of 173°. MTL solution in Tris-NaCl buffer (25 mM Tris, 150 mM NaCl, 25 mM CaCl_2_, pH 8.0) was prepared and filtered using a 0.2 µm pore size filter. Samples were measured in triplicate. A volume of 1 mL of the protein solution under investigation was placed in a glass cuvette and measurements were taken at 25 °C. Aggregation of the MTL solution was induced by raising the concentration of the sample from 0.2 mg/mL to 2 mg/mL and by raising the incubation time of the samples. The results were processed using Microsoft Excel (Microsoft Corporation, Redmond, WA, USA).

### 4.4. Multiple Alignment Analysis of Amino Acid Sequences of Lectins of the Mytilectin Family

Amino acid sequences of Mytilectin family lectins were analyzed by alignment using BLASTp. The complete amino acid sequences of the lectins were obtained from the NCBI database.

### 4.5. In Silico Analysis and Quaternary Structure Prediction

The SWISS-MODEL workspace (http://swissmodel.expasy.org/, accessed on 1 March 2022) was used to search for a template from the PDB for 3D structure prediction. The output was manually filtered to identify lectins showing maximum identity for use as putative templates for homology modeling. The best models were selected for Qmean6 [26] and Z-score [27] using the Swiss Model Validation online tool (http://swissmodel.expasy.org/, accessed on 1 March 2022) [28]. BIOVIA Discovery Studio Visualizer software version 17.2.016349 (Dassault Systemes, Vélizy-Villacoublay, France) was used to visualize the 3D structure of the MTL.

### 4.6. Molecular Modeling of the MTL Structure

The theoretical model of the MTL spatial structure was constructed using the SWISS-MODEL server (https://swissmodel.expasy.org/interactive, accessed on 28 February 2022) containing the template library. As a result, the eight most suitable protein prototypes with an already known crystal structure were found for the target MTL sequence (Table 1). Template search has been performed against the SWISS-MODEL template library (SMTL, last update: 14 September 2022, last included PDB release: 9 September 2022). For each identified template, quality was predicted from the features of the target–template alignment. The templates with the highest quality were then selected for model building. Models were built based on the target–template alignment using ProMod3 (https://openstructure.org/promod3/3.2/, accessed on 3 March 2022) [15]. Coordinates, which are conserved between the target and the template, were copied from the template to the model. Insertions and deletions were remodeled using a fragment library. Side chains were then rebuilt. Finally, the geometry of the resulting model was regularized by force field. The global and per-residue model quality was assessed using the QMEANDisCo (http://swissmodel.expasy.org/, accessed on 1 March 2022) scoring function [18].

## Figures and Tables

**Table 1:** Templates of lectins used to construct a theoretical model of the spatial structure of MTL.

Template (PDB ID)	Seq Identity	Oligo-state	QMEANDisCo	Description
6bfm.1.A	84.00	homo-dimer	0.89 ± 0.05	*M. californianus* lectin
6bfm.1.B	84.00	homo-dimer	0.89 ± 0.05	*M. californianus* lectin
3wmu.1.A	83.78	homo-dimer	0.90 ± 0.05	*M. galloprovincialis* lectin
3wmu.1.B	83.78	homo-dimer	0.90 ± 0.05	*M. galloprovincialis* lectin
5f8s.1.B	83.33	homo-dimer	0.90 ± 0.05	*C. grayanus* lectin
5duy.1.A	83.33	monomer	0.89 ± 0.07	*C. grayanus* lectin
5vbk.1.A	84.00	monomer	0.88 ± 0.07	*M. californianus* lectin
5xg5.1.A	56.62	monomer	0.88 ± 0.07	MITSUBA-1

## Data Availability

Data available on request.
